# Enhancing anti-tumor immune responses through combination therapies: epigenetic drugs and immune checkpoint inhibitors

**DOI:** 10.3389/fimmu.2023.1308264

**Published:** 2023-11-23

**Authors:** Ying Liang, Lingling Wang, Peijun Ma, Dongen Ju, Minggao Zhao, Yun Shi

**Affiliations:** ^1^ Precision Pharmacy and Drug Development Center, Department of Pharmacy, Tangdu Hospital, Fourth Military Medical University, Xi’an, Shaanxi, China; ^2^ Wuchang Hospital Affiliated to Wuhan University of Science and Technology, Wuhan Wuchang Hospital, Wuhan, China; ^3^ Clinical Laboratory, Shanghai Mental Health Center, Shanghai, China; ^4^ Department of Urology, Xijing Hospital, Fourth Military Medical University, Xi’an, Shaanxi, China; ^5^ Department of Immunology and Theranostics, Arthur Riggs Diabetes and Metabolism Research Institute, Beckman Research Institute of the City of Hope, Duarte, CA, United States

**Keywords:** epigenetic regulations, immune cells, T cells, anti-tumor immunity, immune checkpoint inhibitors

## Abstract

Epigenetic mechanisms are processes that affect gene expression and cellular functions without involving changes in the DNA sequence. This abnormal or unstable expression of genes regulated by epigenetics can trigger cancer and other various diseases. The immune cells involved in anti-tumor responses and the immunogenicity of tumors may also be affected by epigenomic changes. This holds significant implications for the development and application of cancer immunotherapy, epigenetic therapy, and their combined treatments in the fight against cancer. We provide an overview of recent research literature focusing on how epigenomic changes in immune cells influence immune cell behavior and function, as well as the immunogenicity of cancer cells. And the combined utilization of epigenetic medications with immune checkpoint inhibitors that focus on immune checkpoint molecules [e.g., Programmed Death 1 (PD-1), Cytotoxic T-Lymphocyte-Associated Protein 4 (CTLA-4), T cell Immunoglobulin and Mucin Domain (TIM-3), Lymphocyte Activation Gene-3 (LAG-3)] present in immune cells and stromal cells associated with tumors. We highlight the potential of small-molecule inhibitors targeting epigenetic regulators to amplify anti-tumor immune responses. Moreover, we discuss how to leverage the intricate relationship between cancer epigenetics and cancer immunology to create treatment regimens that integrate epigenetic therapies with immunotherapies.

## Introduction

Epigenetic modifications are heritable and alters gene expression by changing chromatin structure and modifications, like DNA methylation, histone modifications, and non-coding RNA regulations (1). During the 1980s, DNA hypomethylation at genome-wide level was firstly discovered as a critical feature of cancer cells; meanwhile DNA hypermethylation can result to tumor suppressor genes (TSGs) silence (2). Another research found that hypermethylation of cytosine residues, as well as somatic mutations in *TET2*, *DNMT3B*, *IDH1*, and *BRAF* are exhibited in approximately 22% of tumors (3). MicroRNAs (miRNAs) are small non-coding RNAs (ncRNAs) spanning 19~22 nucleotides, which regulate gene expression by inhibiting or degrading its mRNA in a sequence-dependent way. Fabbri et al., reported the first evidence for miR-29 family involvement in directly targeting two DNA methyltransferases: DNMT3a/DNMT3b. Further, inhibiting miR-29s or DNMTs restored epigenetically silenced TSGs expression (4). Others’ findings identified Lrg1, Vegf-a, and IL-10 can be activated by histone H3K18 lactylation as reparative genes via combination of CUT&Tag and RNA-sequencing profiling, which is critical to immune homeostasis for post-myocardial infarction (5). This review will focus on epigenetic modifications during tumorigenesis and will emphasize opportunities to develop therapeutic strategies that target these modifications in cancer.

The accumulation of epigenetic changes is pivotal in cancer development (6–8). Various tumor-specific alterations create neoantigens, and epigenetic changes can contribute to the reactivation of genes that are typically restricted to immune-privileged stages, leading to their expression (9). Both tumor neoantigens and autoantigens can be immunogenic, eliciting an immune response in the body (10). Over the past few decades, significant attention has been directed towards the development of epigenetic therapies as anticancer agents, owing to their direct impact on cancer cells. recent studies have not only shed light on how epigenetic mechanisms contribute to immune evasion but have also unraveled the role of epigenetic drugs in modulating immune pathways to enhance immune recognition and promote immunogenicity (11–13). A comprehensive understanding of the role played by epigenetic regulatory mechanisms in cancer immunity is crucial to fully harnessing the potential of epigenetic drugs.

Immunotherapy is considered the fourth major technique in dealing with cancers, following surgery, radiotherapy, and chemotherapy. The fundamental objective of immunotherapy is to reinitiate and maintain the tumor-immune cycle, restoring the body’s natural anti-tumor immune response. By enhancing the immune system’s capacity to identify and focus on cancerous cells, immunotherapy aims to control and eliminate tumors. Among immunotherapies, immune checkpoint inhibitors have undergone rapid clinical development and have been widely utilized clinically. Up to now, the U.S. Food and Drug Administration (FDA) has authorized the use of a pair of anti-CTLA-4 antibodies, along with three anti-PD-1 antibodies and three anti-PD-L1 antibodies for treatment of more than a dozen different cancers (14, 15); Nonetheless, a considerable number of patients continue to encounter restricted advantages concerning their response to therapy and overall survival. In addition, epigenetic mechanisms are also involved in the development of tumors and tumor immunogenicity. Epigenetic therapies have shown remarkable clinical efficacy in hematological cancers, yet their efficacy in solid tumors has been limited (16, 17). In recent years, extensive research has revealed a significant breakthrough in tumor treatment, highlighting the effectiveness of a combined approach involving immunosuppressants and epigenetic drugs. Efforts must be directed towards the development of remedies aimed at investigating combination therapies, with the ultimate goal of improving response rates.

## Epigenetic regulations and epidrugs in cancer

**Figure 1:**
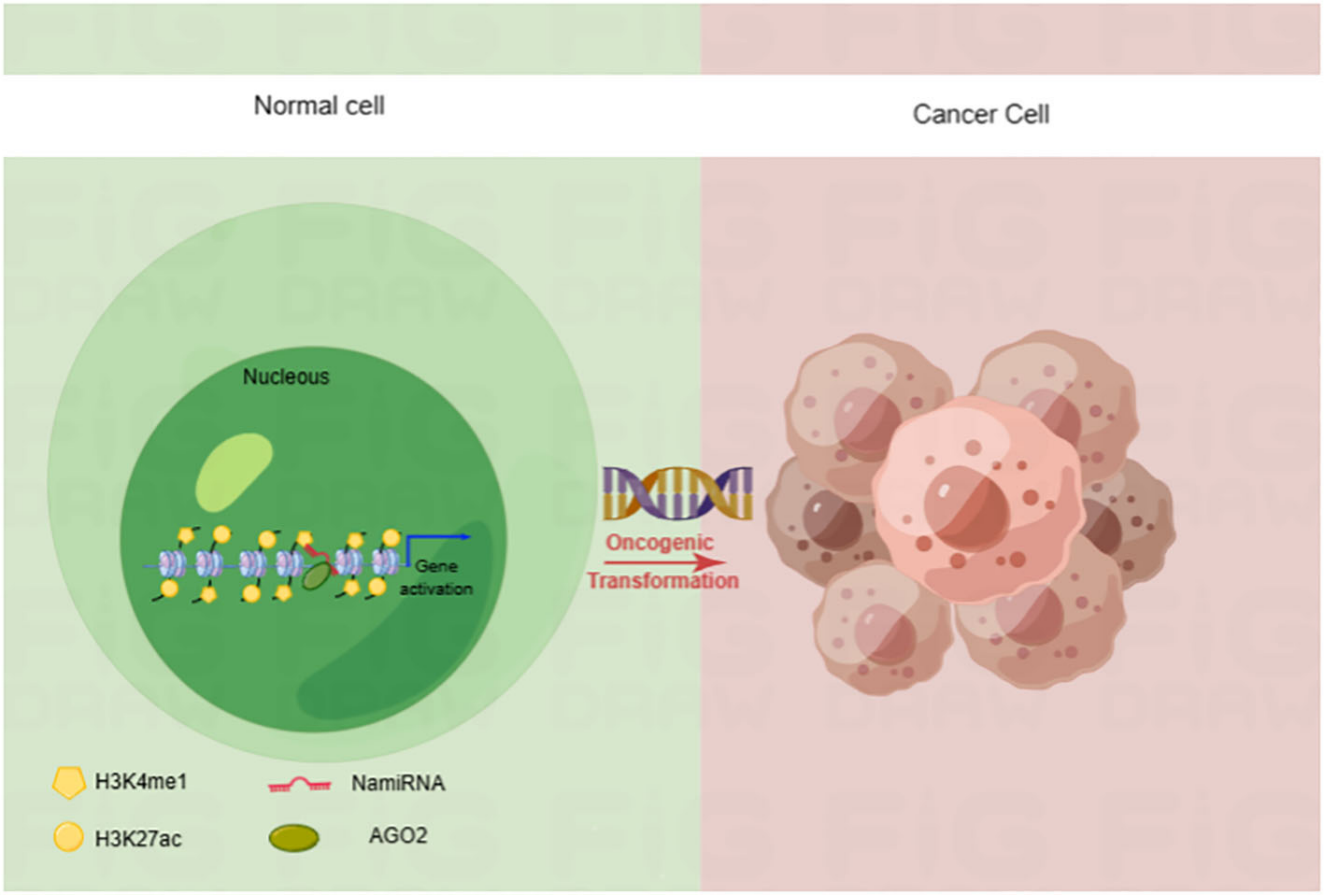
NamiRNA plays positive regulations on gene transcription through enhancers in nucleus. NamiRNAs exert positive roles on target gene expression to promote or block cancer progression. H3K4me1 and H3K27ac are enhancer markers.

Epigenetic dysregulation is a common contribution to tumorigenesis. In recent years, our lab poured great energy into a systematic analysis of them to comprehensively explore the function of miRNAs (18). We analyzed the relationship between the location of human miRNA loci and the enrichment of enhancer markers Histone 3 Lysine 27 acetylation (H3K27ac) and Histone 3 Lysine 4 monomethylation (H3K4me1). Finally, we found that many miRNA precursor loci overlapped with the cognate enhancer regions enriched for H3K27ac and H3K4me1, such as miR-24-1, miR-339, and miR-3179. Interestingly, enhancer-associated miR-24-1 was also demonstrated to be able to activate its neighboring genes. Such enhancer-associated miRNAs that activate gene expression are known as nuclear activating miRNAs (NamiRNAs) (19). In the case of miR-339 and miR-24-1, in addition to exerting a positive regulatory function on neighboring gene activation via AGO2 proteins, these NamiRNAs are also involved in tumorigenesis (20, 21) (**Figure 1**). Therefore, NamiRNAs may be promising epigenetic targets for cancer therapy in the future.

The quick development of epidrugs over the past two decades brings new prospects to cancer therapy (22). Before the year of 2020, the range of FDA-approved epidrugs was limited, comprising solely of four pan-Histone deacetylase inhibitor (HDACi) and two DNA methyltransferase inhibitors (DNMTi). These treatments were specifically approved to target hematopoietic malignancies, including T-cell lymphoma, and myelodysplastic syndromes. Chidamide (also known as HBI-8000), an additional HDACi, received approval from the Chinese Food and Drug Administration for the treatment of peripheral T-cell lymphoma (23). Drugs like hydralazine, procaine and procainamide, originally approved by the FDA for hypertension treatment, local anesthesia and cardiac arrhythmia, respectively, have also demonstrated DNMTi activity (24). Likewise, valproic acid, an FDA-approved drug primarily used for treating seizures, also possess HDACi properties. In January 2020, a significant milestone was achieved with the approval of tazemetostat, an inhibitor targeting KMT6A (EZH2), for the treatment of epithelioid sarcoma. This marked a groundbreaking advancement as it became the first approved inhibitor of histone ‘writers’ and the first epidrug to demonstrate efficacy in treating solid tumors (25).

## Epigenetic regulations in immune cells

**Figure 2:**
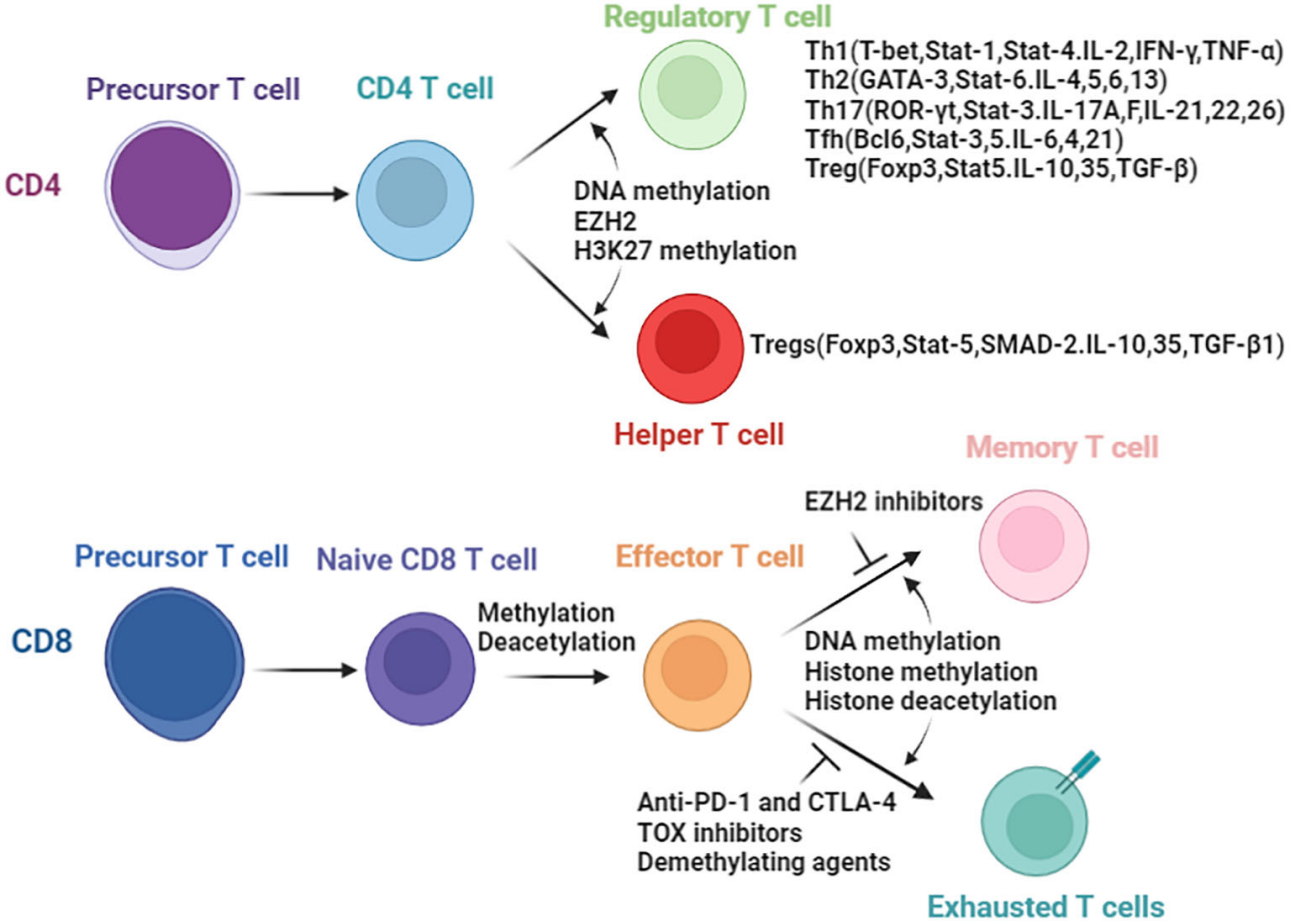
Epigenetic enzymes involved in the biology of T cells. Differentiation of CD4 T cells and CD8 T cells are controlled by epigenetic modifications. In parenthesis are the transcript factors and secreted cytokines resulting directly or indirectly due to epigenetic changes.

Understanding the influence of epigenetics on immune cells are important in tumor immunotherapy and epigenetic therapy. Epigenetic regulations play a key role in the, differentiation, development and function of various immunocyte lineages (26–28). In this section, we aim to emphasize the role of epigenetic mechanisms in governing the fate and development of immune cell subsets during anti-tumor immune responses. We specifically focus on CD4^+,^CD8^+^ T lymphocytes and natural killer (NK) cells, highlighting the impact of epigenetic regulations. Herein, we try to comprehensively review the whole picture of the crosstalk between immunity and epigenetic regulation (**Figure 2**), and summarize the epigenetic regulations and outcomes in CD4^+^ and CD8^+^ T cells.

### Epigenetic mechanism exerts critical roles in CD4^+^ T cells

**Figure 3:**
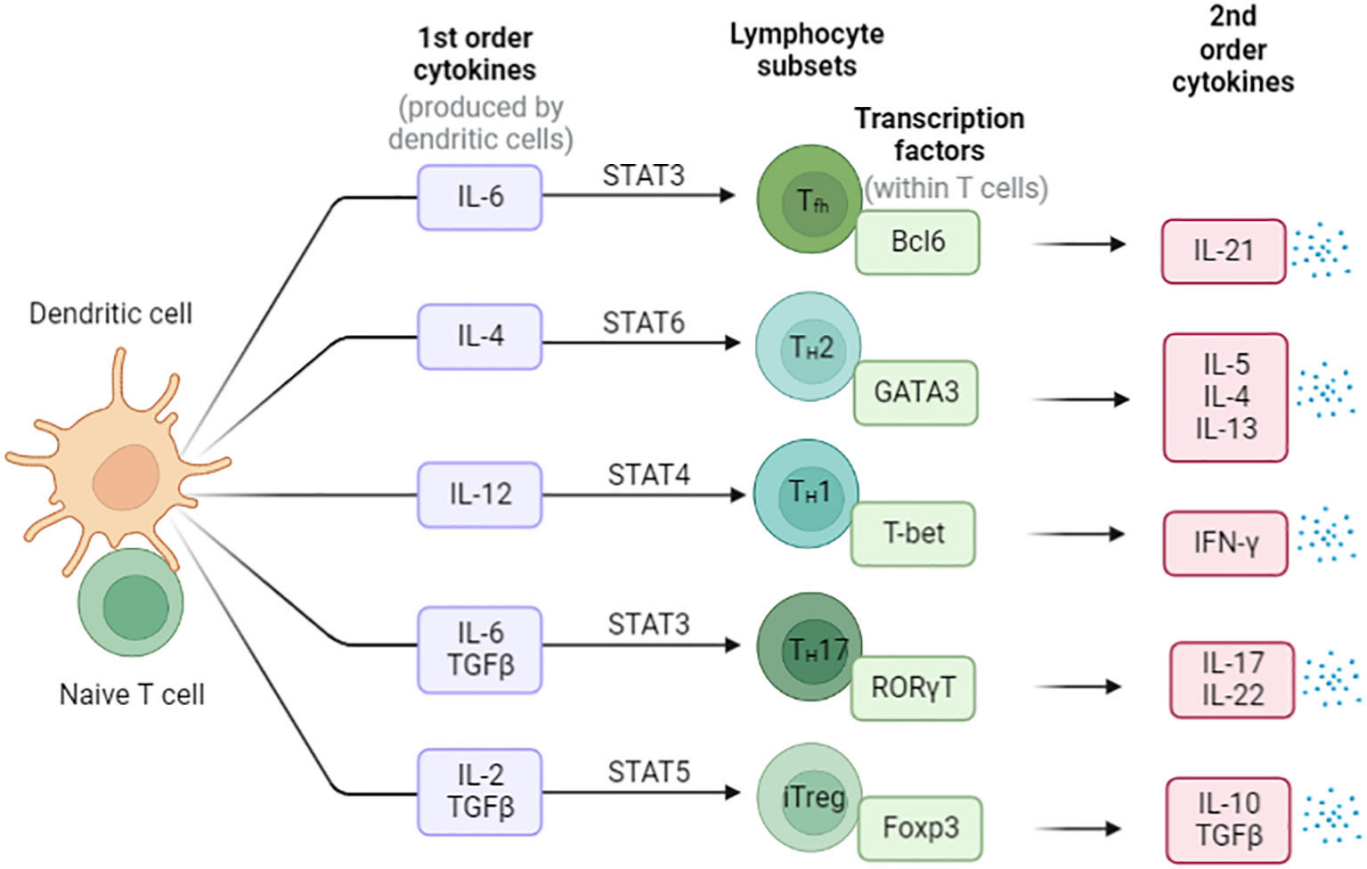
The differentiation of Naïve CD4^+^ T cells in the periphery. Cytokines direct the differentiation of these different types of helper T cells, and the major mode of helper T cell effector function is selective cytokine secretion. These T cell subsets are characterized by the expression of master transcription factors and secretion of signature cytokines.

CD4^+^ T cells play a variety functions in the immune system and differentiate into several subsets of effector T cells. Including T helper 1 (Th1) cells, T helper 2 (Th2) cells, T helper 17 (Th17) cells, T follicular helper (Tfh) cells, and regulatory T (Treg) cells (29) (**Figure 3**). Th1 cells secrete gamma interferon (IFN-γ) and tumor necrosis factor alpha (TNF-α) and are important for resistance to intracellular pathogens and cancer, whereas Th2 cells secrete cytokines interleukin-4 (IL-4), IL-5, IL-9 and IL-13, which stimulate B-cell antibody production and resist other extracellular pathogens (30–32). Th17 cells produce the cytokines IL-17A, IL-17F, IL-21 and IL-22 that direct neutrophil-mediated attack against extracellular bacteria infections (33). T_reg_ cells secrete Inhibitory cytokines IL-10 (34) and transforming growth factor-β (TGF-β) (35–38), which suppress the activity of a variety of immune cells and play critical roles in maintaining immune homeostasis.

Epigenetic mechanisms play important roles in the differentiation and functions of T cells (39, 40). Enhancer of zeste homology 2 (EZH2) is a histone H3K27 methyltransferase. Deficiency of EZH2 was found to specifically reduce the differentiation and plasticity of Th1 and Th2 cell depend on loss of H3K27me3 at genes encoding T-bet and Gata3 for Th1 and Th2 cells, respectively (41). Up to 10% of the mouse genome is made up of endogenous retrovirus (ERV) sequences (42). *Véronique Adoue et al.* showed that the histone methyltransferase SET domain bifurcated 1 (SETDB1) deposits the repressive H3K9me3 mark at ERVs in Th2 cells. The level of chromatin by the SETDB1-dependent deposition of H3K9me3 at ERVs control Th2 cell stability (43). For Th2 cells, the acquisition and maintenance of the Th2 cell identity relies on epigenetic modifications that establish a stable gene expression program that supports Th2-specific cytokine production and effector responses (44).

As for Th17 cells, Epigenetic modifications have emerged as important mechanisms for regulating the production of IL-17 during Th17 cell differentiation (45, 46). Reseachers uncovered that the enrichment of permissive histone modifications such as H3K4me3 and DNA demethylation in the promoters of cytokine and genes such as *IL17a, IL17f, IL21, IL23r*, and *Rorc* in TH17 cells. CXXC finger protein 1 (Cxxc1) is an epigenetic regulator, Feng Lin et al. showed Cxxc1 is critical for T-cell intrathymic development and through regulating H3K4 trimethylation, Cxxc1 deficiency decreased the generation of TH17 cells and prevented their differentiation into T_reg_ cells (47). Cxxc1-dependent H3K4me3 plays a critical role in thymocyte development, phagocytosis, and the bactericidal activity of macrophages (48, 49).

### Epigenetic regulation of CD8^+^ T cells

CD8^+^ T cells play important role in anti-tumor immunotherapies and pathogen clearance and resist re-infection through long-lived immune memory T cells (50). Similar to the differentiation of naïve CD4^+^ cells, naive CD8^+^ T cells differentiate into a variety of memory and effector cell types under stimulation.The epigenetic regulation of CD8^+^ T cells has been extensively investigated (51).

T cell metabolism is influenced by environmental nutrient availability, which impacting T cell function. Ketone bodies (KBs) include β-hydroxybutyrate (βOHB) and acetoacetate (AcAc). Katarzyna et al. showed that CD8^+^ Teff cells prefer to use KBs rather than glucose to fuel the tricarboxylic acid (TCA) cycle both *in vitro* and *in vivo*. βOHB increased CD8^+^ Teff cells cytokine production and cytolytic activity responses through effects on histone acetylation at effector gene loci (52). The activation process of CD8^+^ T cell involves dynamic changes in DNA and histone modifications. Eomesodermin (EOMES) is a key transcription factor in regulating the differentiation of naïve CD8^+^ T cells into effector and memory CD8^+^ T cells (53). Perforin (*PRF1*), granzyme B (*GZMB*), and Gamma interferon *(IFNG*) are the target genes of EOMES, and promotes effector function in CD8^+^ T cells (54–56). Yasuto Araki et al. showed that compared with naive CD8^+^ T cells, the levels of H3K9Ac were significant higher in the *EOMES*, *PRF1*, and *GZMB* loci in memory CD8^+^ T cells.

### Epigenetic regulation of T_reg_ cells

**Figure 4:**
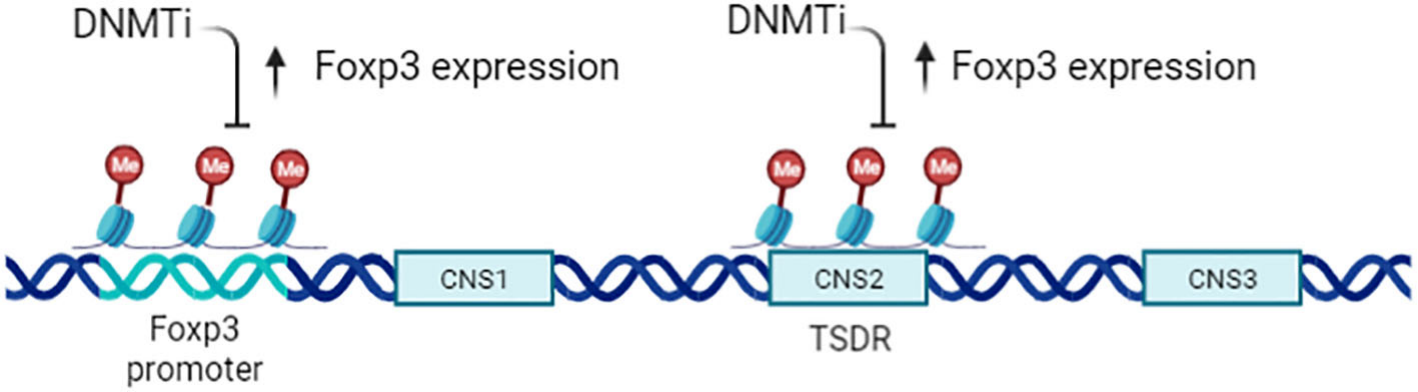
Epigenetic regulation of CD4^+^CD25^+^ T_regs_. Expression of Foxp3 is tightly controlled by the methylation status of the gene, particularly the T_reg_ -specific demethylation region (TSDR;CNS2). A DNMTi can enhance Foxp3 expression but does not increase T_reg_ function, owing to the altered epigenetic state of Foxp3 target genes.

T_reg_ cells inhibit inflammatory effector T cells and play an opposite effect in anti-tumor immunity. The generation, maintenance, and function of CD4^+^ CD25^+^ T_reg_ cells is critically dependent on transcription factor forkhead box P3 (Foxp3) (36, 57, 58), and epigenetic regulation is important for Foxp3 activation (59–61). nT_reg_ exhibit a specific CpG hypomethylation pattern which is independent of Foxp3 expression, what’s more, Foxp3 induction and CpG hypomethylation are both required in the development of T_reg_ cell (46). Furthermore,T_reg_ exhibit specific DNA methylation signature that help distinguish T_reg_ from T_conv_ cells (62), especially in the Foxp3 gene region.There are at least three conserved non-coding sequence (CNS1–CNS3) *cis-elements* at the foxp3 gene locus (**Figure 4**), the methylation status of which determine the expression and stability of Foxp3 (63, 64). (Conserved non-coding sequence 22) CNS2 is a CpG-rich Foxp3 intronic cis-element specifically demethylated in mature T_reg_, helps prevents autoimmunity and maintain immune homeostasis, thus CNS2 play an important role in the stability and function of T_regs_ (65, 66). The demethylation of the CNS2 is dependent on the activity of Ten-eleven translocation (TET) family members and induction of TET activity has been recognized as a mechanism for promoting the function of T_reg_ cells in autoimmune diseases (67, 68). Mice conditionally deficient for Tet2/Tet3 in T_regs_ develop inflammatory disease, and the T_reg_ cells gene signatures are altered in these mice, indicating that Tet2/3 are master regulators that stabilize T_reg_ cells identity and immune homeostasis (69).

### Epigenetic regulation of NK cells

Natural killer (NK) cells have been classified as a type of lymphocyte, NK cells are generally considered components of the innate system. These cells have a high cytolytic capacity, unlike cytotoxic T cells, can induce tumor cell death directly and are imperative for tumor immunotherapy (70).

The tumor immune escape of NK cells has multiple molecular mechanisms. These include some upregulation of inhibitory receptors on NK cells such as HLA-E and HLA-G (71, 72), the secretion of chemokines and cytokines like IL-10, transforming growth factor β (TGF-β), CXCL9 and prostaglandin E2 (73, 74), as well as the expression of inhibitory receptors including PD-1,CTLA-4,TIM-3,and LAG3 (75–77). DNA and histone methylation have been implicated in the downregulation of NK inhibitory receptor CD94/NK group 2 member A (NKG2A). What’s more, the methylation of H3K27, mediated by EZH2, leads to the suppress NK cells via ligation of the natural killer group 2D (NKG2D) receptor. Moreover, H3K27 methylation also induce silencing of IL-15, CD122 NKG2D receptor proteins, which consequently suppress NK cell expansion and decreases their cytotoxic targeting of tumor cells (78–80). The cytotoxicity of NK cells can also be suppressed by H3K9 and H3K4 methylation, leading to the downregulation of the NKG2D receptor. However, when the methyltransferases responsible for these methylation processes were inhibited, the expression level of the ligands increases, resulting in enhanced anti-tumor cytotoxicity of NK cells (81–84).

## Recent advances in cancer immune therapy

**Figure 5:**
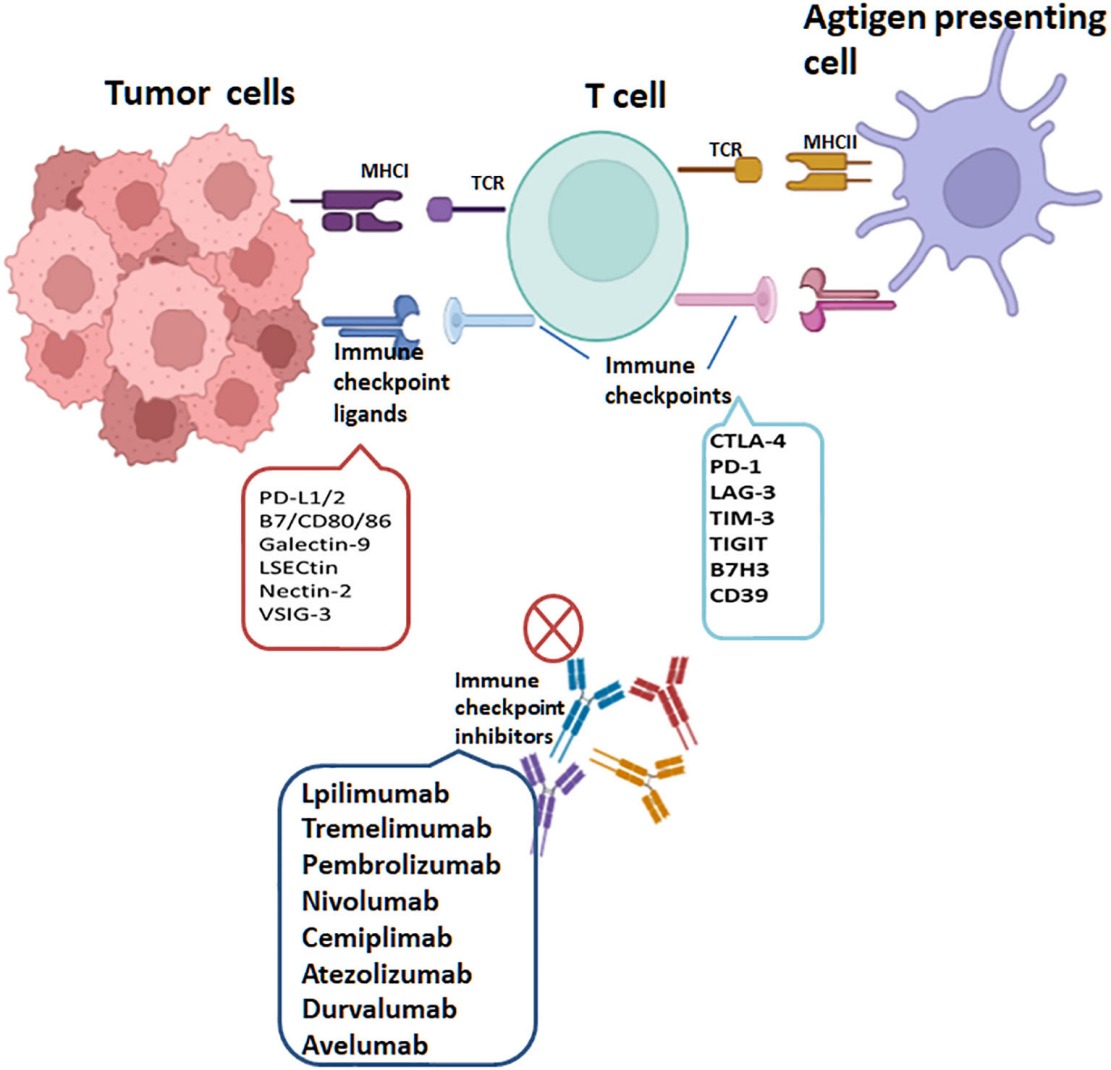
T cells are activated through MHC class I expressed on tumor cells or MHC class II molecules expressed on antigen-presenting cells, recognized by T-Cell Receptor (TCR). PD-1, PD-L1, CTLA-4, LAG-3, TIM-3, TIGIT, CD39 and B7H3 are targets of immune checkpoints (ICs). Interactions of ICs with their ligands on tumor cells lead to T cell inactivation. The immune checkpoint inhibitors(ICIs) currently approved through the FDA include lpilimumab and tremelimumab targeting CTLA-4, pembrolizumab, cemiplimab and nivolumab targeting PD-1 and atezolizumab, avelumab and durvalumab targeting PD-L1.

With extensive research on tumor immunogenicity and immune cells, cancer treatment has witnessed a notable advancement with the emergence of tumor immunotherapy, specifically immune checkpoint inhibitors (ICIs). It has revolutionized the field of oncology and shown remarkable clinical efficacy in certain types of cancer, especially in treating solid tumors. Presently, ICIs targeting three distinct molecules, namely CTLA-4, PD-1, and PD-L1, have gained widespread usage. CTLA-4 exerts inhibitory actions on T cells through multiple mechanisms. Firstly, it initiates receptor signaling that recruits phosphatases, subsequently suppressing the activity of essential transcription factors crucial for T cell function, like NF-κB, AP-1, and NFAT. Moreover, CTLA-4’s competition with CD28 for ligand binding diminishes the co-stimulatory signals accessible to T cells, resulting in a continued suppression of their activation (85). Additionally, research has demonstrated that CTLA-4 also contributes to the augmentation of T_regs_’ function and activity. It promotes the suppressive capacity of T_regs_, thereby exerting an additional regulatory effect on immune responses (14, 86). The effectiveness of inhibiting CTLA-4 has been extensively validated in a diverse range of tumors, both in preclinical studies and clinical trials. Notably, it obtained FDA approval in March 2011 for treating advanced melanoma. Additionally, ipilimumab, an anti-CTLA-4 antibody, is presently being studied as a possible therapeutic option for a range of cancer types, including prostate cancer, non-small-cell lung carcinoma (NSCLC), renal cell carcinoma (RCC), and other malignancies (87). PD-1 is predominantly expressed during the later phases of T cell activation. It can also be found on the surface of B cells and macrophages. Contrastingly, CTLA-4 primarily regulates the extent of early activation in both naïve and memory T cells. These distinct expression patterns and functions highlight the complementary roles played by PD-1 and CTLA-4 in modulating immune responses at different stages. The interplay of PD-1 with its counterpart PD-L1 initiates a signaling cascade that can induce T cell apoptosis, leading to a state of T cell exhaustion. This exhaustion impairs the functionality and responsiveness of T cells, ultimately aiding in tumor cells’ evasion of the immune response (88). In the context of immunotherapy, PD-1/PD-L1 antibodies function by interrupting the PD-1 and PD-L1 interaction. This disruption enables T cells to undergo reactivation, proliferation and enhances their capacity to identify and eradicate tumor cells. PD-1 blockade primarily operates by attenuating the proximal T cell receptor (TCR) signaling pathway, thereby restoring the depleted CD8^+^ effector T cell population. The reinvigoration of T cell function plays a vital role in bolstering the anti-tumor immune response, effectively combating tumor growth. In 2014, the FDA granted approval for two anti-PD-1 antibodies, namely pembrolizumab and nivolumab, to be used in treating advanced melanoma. Furthermore, over the recent years, the FDA has approved three anti-PD-L1 antibodies—namely atezolizumab, avelumab and durvalumab—for treating various types of cancer. In addition, an expanding array of checkpoint receptors and ligands are being targeted in clinical settings, adding to the list of potential therapeutic options. These include TIM3, B7H3, LAG3, CD39, TIGIT, IDO-1, CD73, adenosine A2A receptor, among others (**Figure 5**). The exploration and development of therapies targeting these checkpoints highlight the expanding landscape of immune checkpoint inhibition and the potential for new treatment options in various cancers (75, 89–93). The efficacy of various combinations-immune checkpoint inhibitors is being investigated currently in clinical trials.

## Epigenetic mechanisms in ICI resistance

While ICIs have shown remarkable efficacy in treating certain cancers, a subset of patients may experience primary or acquired resistance to these therapies. Additionally, the use of ICIs can give rise to immune-related adverse events (IRAEs) impacting multiple organs (94). As a result, in many tumor indications the objective response rate (ORR) to ICIs can be less than 40% (95). The primary resistance mechanism to ICIs mainly includes: 1) a diminished expression of PD-L1 on tumor cells; 2) diminished response to ICIs due to tumor-intrinsic factors, such as genetic alterations within tumor cells that impact antigen presentation, interferon signaling, or downstream signaling pathways; 3) the loss of the IFN-γ signaling pathway; and 4) other factors that influence the enteric microbiome, effector T-cell infiltration in the tumor microenvironment (TME) and T-cell exhaustion. Another crucial mechanism that promotes resistance to ICIs involves epigenetic modulation, primarily encompassing changes in histone marks and chromatin structure, alterations in DNA methylation and dysregulation of regulatory RNA expression levels (96–98). For instance, HDACi can increase the levels of tumor antigens and reactivate proapoptotic genes, thereby sensitizing tumor cells to immune-mediated destruction (99). Histone methyltransferases (HMT) such as EZH2 have been identified as playing a role in the regulation of immune checkpoint molecules. HMTs have the capacity to inhibit the expression of PD-L1 by increasing H3K27me3 levels on the promoters of CD274 (PD-L1 gene), potentially reducing the immune evasion capabilities of tumor cells (100). In certain human melanoma cell lines, DNA hypomethylation has been associated with constant elevation of cytokines like IL-6 and VEGF. This dysregulated cytokine expression may contribute to resistance against immunotherapy (28). MiR-214, miR-568 and miR-126 contribute to T-cell exhaustion by influencing the development and function of T_regs_. These microRNAs are also involved in downregulating cytotoxic T lymphocyte (CTL) activity (101).

## Combinatorial therapy with epigenetic drugs and ICIs

Epigenetic targeting agents as a novel class of immune modulators have shown significant efficacy in hematological malignancies (102). However, their efficacy in solid tumors is constrained. Given that both epigenetic therapy and immune checkpoint blockade target the tumor microenvironment, combining ICIs with epigenetic modulators shows great promise in overcoming various cases of ICI resistance. This combinatory approach has the potential not only to generate synergistic effects but also mitigate adverse effects and deter the emergence of drug resistance. Some clinical trials and structures of the chemical compounds[ (103–105)] are underway or completed are listed below (**Table 1**).

**Table 1:** Epigenetic strategies synergize with PD-L1/PD-1 targeted cancer immunotherapies.

Epigenetic drug	Structures of the chemical compounds	Immune checkpoint inhibitors	Status/Phase	Cancer type	Trial ID
Chidamide (HDACi)	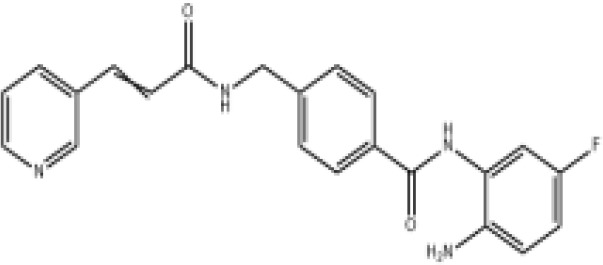	Toripalimab	Recruiting/Ib/II	Advanced Cervical Cancer	NCT04651127
Entinostat (HDACi)		Atezolizumab Bevacizumab	Suspended/I/II	Advanced Renal Cell Carcinoma	NCT03024437
Chidamide (HDACi)	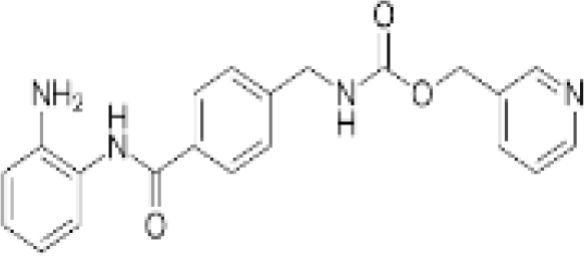	Sintilimab	Not yet recruiting/II	Refractory and Relapsed AITL	NCT04831710
Chidamide (HDACi)	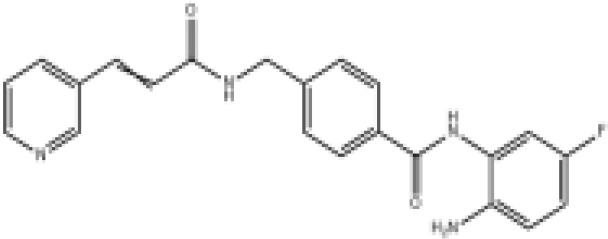	Sintilimab	Recruiting/II	Peripheral T Cell Lymphoma	NCT04512534
Chidamide (HDACi)	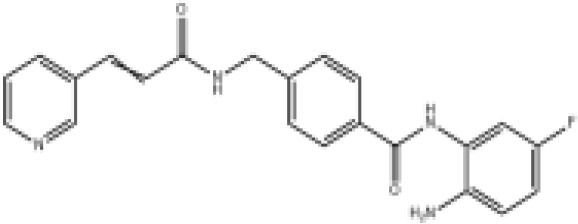	Immune Checkpoint Inhibitors	Recruiting/I/II	R/R NHL and Advanced Solid Tumors	NCT05320640
Decitabine (DNMTi)	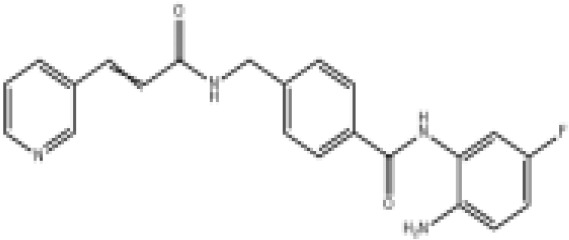				
Mocetinostat (HDACi)	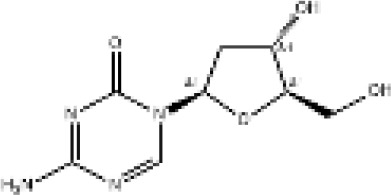	Durvalumab	Terminated HasResults/I/II	Advanced Solid Tumors and NSCLC	NCT02805660
Entinostat (HDACi)	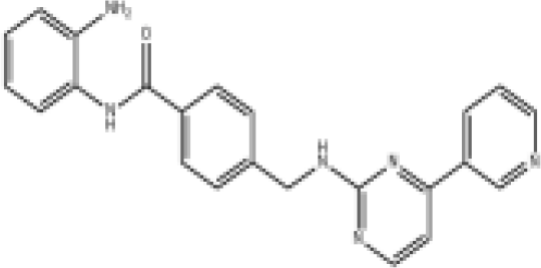	Pembrolizumab	Active, not recruiting/Ib/II	NSCLC, Melanoma,and Colorectal Cancer	NCT02437136
Guadecitabine (DNMTi)	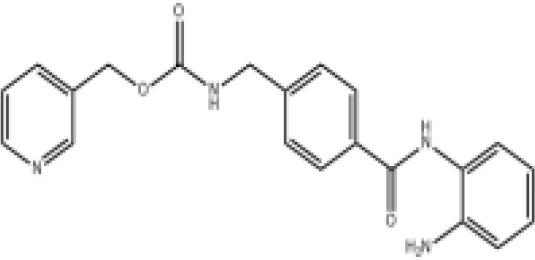	Atezolizumab	Active,not recruiting/I/II	Advanced MDS,Refractory or Relapsed CML	NCT02935361
Decitabine(DNMT)	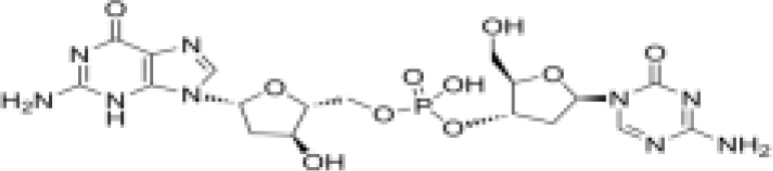	PDR001 MBG453	Active,not recruiting/I	AML or High-Risk MDS	NCT03066648
Belinostat (HDACi)	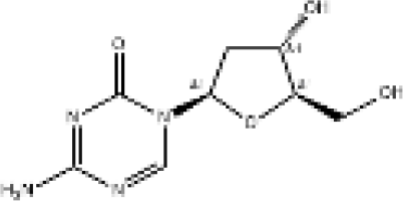	Tremelimumab Durvalumab	Recruiting/I	ARID1A Mutated Metastatic or Unresectable, Locally Advanced Urothelial Carcinoma	NCT05154994
Entinostat(HDACi)	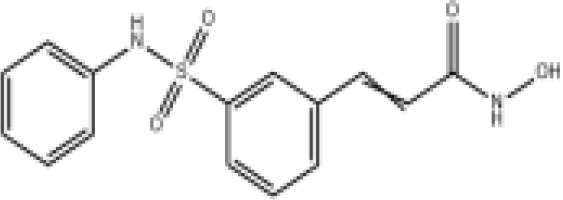	Nivolumab Ipilimumab	Active,not recruiting/I	Solid Tumors That Are Metastatic or Cannot Be Moved by Surgery or Locally Advanced or Metastatic HER2-Negative Breast Cancer	NCT02453620
Chidamide (HDACi)	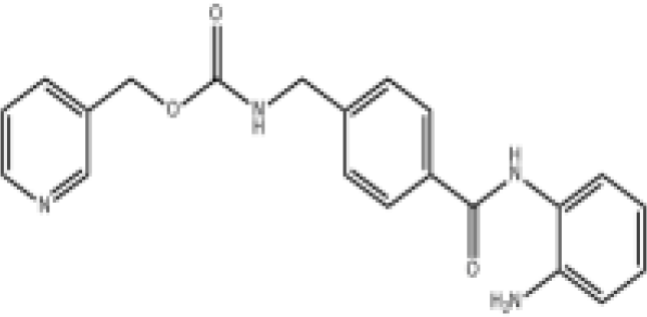	Sintilimab	Completed/Ib/II	Refractory and Relapsed ENKTCL	NCT03820596
Oral 5-Azacitidine	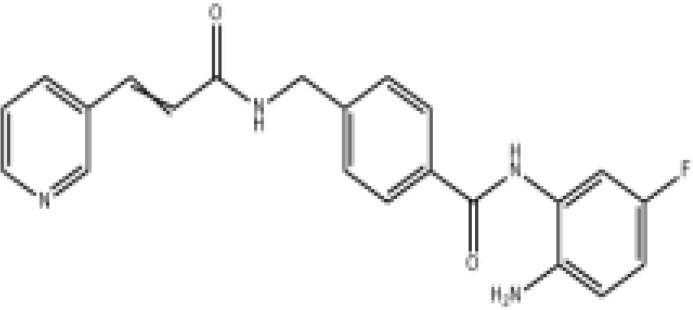	Durvalumab	Recruiting/I/IIa	Lymphoma	NCT03161223
Romidepsin (HDACi)	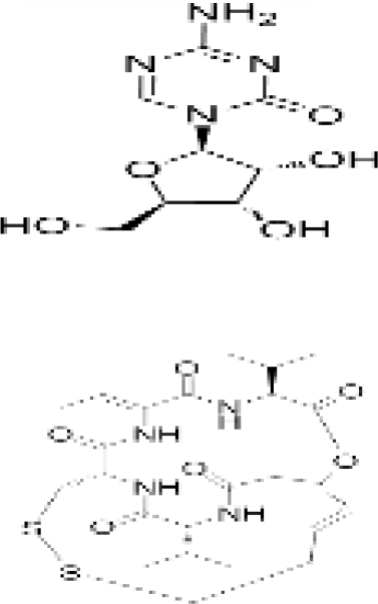				
ZEN003694 (BETi)	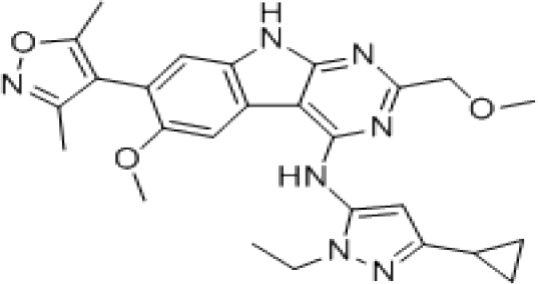	Nivolumab	Recruiting/I/Ib	Solid Tumors	NCT04840589
ZEN003694 (BETi)	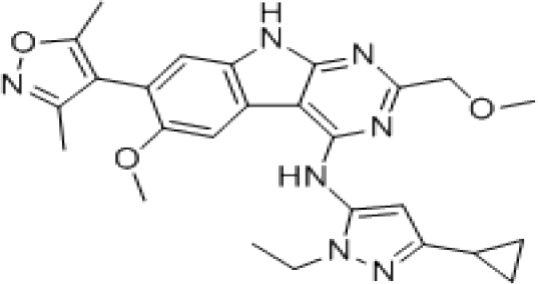	Pembrolizumab	Recruiting/Ib	Advanced Triple-Negative Breast	NCT05422794

### DNA methyltransferase inhibitors and ICIs

DNMTi holds a pivotal position during the outset of the immune cycle, particularly by augmenting the immunogenicity of tumors and facilitating immune recognition through the re-expression of tumor-associated antigens. Additionally, the upregulation of MHC class I and II molecules by DNMTi has been observed in numerous cancer types (106). In individuals with melanoma, global DNA hypermethylation is often linked to decreased expression levels of PD-L1. Research has shown that DNA hypomethylation can enhance the expression of CTLA-4, PD-L1, PD-1, and TIGIT in human colorectal cancer (107). In a murine ovarian cancer model, the administration of decitabine in combination with anti-CTLA-4 therapy led to increased efficacy and prolonged CD8^+^ tumor-infiltrating lymphocytes (TILs) response (108). Peng et al. (109) further demonstrated that inhibitors targeting DNMT1 and EZH2 have the ability to reawaken the production of T-helper 1 (Th1) chemokines, specifically CXCL9 and CXCL10, in human ovarian cancer. This reactivation resulted in an augmented infiltration of effector T cells into the tumor, subsequently decelerating tumor progression and enhancing the therapeutic effectiveness of anti-PD-L1 treatment. It is worth noting that, the DNA methylation status of LAG-3 is correlated with the expression levels of this immune checkpoint molecule in both tumor cells and immune cells in clear cell renal cell carcinoma (ccRCC) (110); this phenomenon significantly influences the destiny of immune cell infiltration within the tumor microenvironment.

### Histone regulators and ICIs

The key histone modifications implicated in the cancer process primarily involve HDACs, HMTs like EZH2, and the Histone Reader Proteins of the BET (Bromodomain and Extra-Terminal) family. HDAC inhibitors have shown encouraging outcomes in overcoming ICI resistance in diverse cancer cell lines (110, 111), and in patients with metastatic non-small cell lung cancer (NSCLC), the use of HDAC inhibitor entinostat increased the PD-1 blockade immunotherapy response and facilitated heightened T cell infiltration (112). In a separate study, entinostat was found to enhance the therapeutic effectiveness of anti-PD-1 treatment by inhibiting myeloid-derived suppressor cells (MDSCs) in lung and kidney cancer mouse models (113). These findings underscore the potential of HDAC inhibitors in modulating the immune microenvironment and enhancing the efficacy of immunotherapies in certain cancer types. Multiple arguments support the association between EZH2 expression and tumor immunogenicity, indicating that interfering with EZH2 expression may have an impact on the response to ICIs. Indeed, another study has reported that inactivating EZH2 can reverse resistance to anti-CTLA-4 and IL-2 immunotherapies and inhibit the growth of melanoma (114). In the context of hepatocellular carcinoma, EZH2-mediated upregulation of H3K27me3 levels on the CD274 promoter, which encodes PD-L1, leads to a reduction in the expression of PD-L1 (100). Targeting EZH2 has been proposed as a potential strategy to enhance tumor immunogenicity. Additional investigation is warranted to comprehensively grasp the intricate interplay between EZH2, tumor immunogenicity, and the response to ICIs. However, the evidence thus far suggests that targeting EZH2 may represent a promising approach to enhance the effectiveness of immune-based cancer therapies. Indeed, the BET family are known to bind to acetylated histones and modulate immune function-related gene transcription. Inhibition of BET family members has demonstrated a decrease in PD-L1 expression, cytokine production, and nuclear factor-κB (NF-kB) activity in cancer cells (97, 101, 115). JQ1, a potent BET inhibitor, effectively boosts the body’s anti-tumor immune response by enhancing the cytotoxicity of CTLs while simultaneously reducing the infiltration of T_regs_. JQ1 can not only coordinate the anti-tumor effect of PD-1 but also regulate PD-L1 and promote the expression of MHC-I molecules in colorectal cancer (CRC) cells (116). However, the exact mechanism underlying this regulation is not yet fully understood and needs further study. The modulation of BET proteins provides a potential therapeutic avenue for targeting the immune response in cancer and other immune-related diseases (117, 118). Inhibitors targeting the BET family are currently under investigation for their potential to augment the effectiveness of immunotherapies and surmount resistance to immune checkpoint blockade across diverse cancer types.

### miRNAs and ICIs

**Figure 6:**
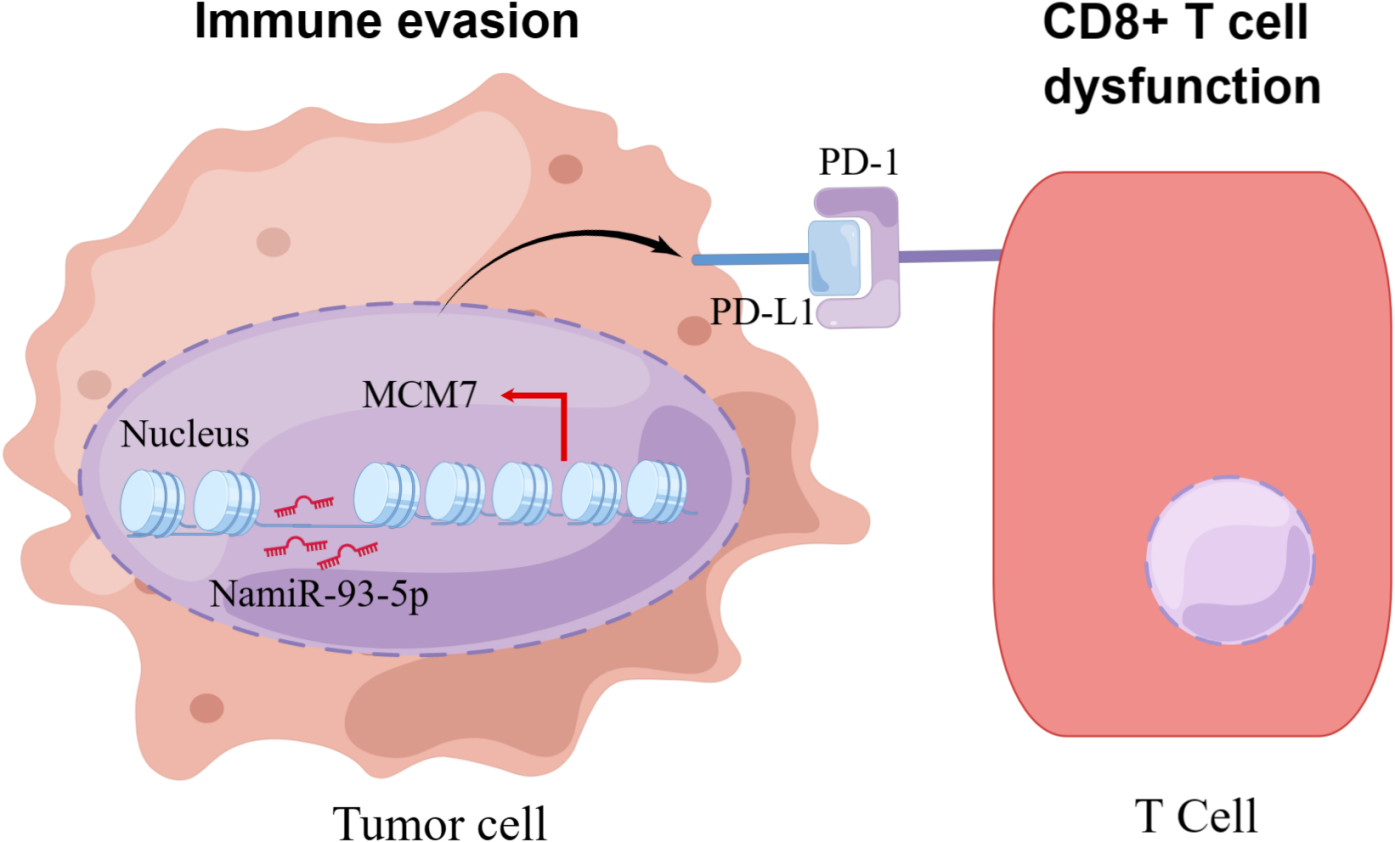
The schematic diagram of how NamiRNA-93-5p activates *MCM7* to promote immune escape. In kidney cancer cells, NamiRNA-93-5p binds to the enhancer of *MCM7* to activate its expression in the nucleus, thus inducing activation of PD-1/PD-L1 pathway and dysfunction of CD8^+^ T cells, and finally contributes to immune escape.

MiRNAs are also a critical component of epigenetic modifications. MiRNAs, both directly and indirectly, regulate the expression of PD-L1 and several other immune checkpoints. These miRNAs exert their regulatory influence by either binding directly to the PD-L1 mRNA or targeting other molecules involved in the signaling pathways that control PD-L1 expression. At least two independent studies noted that miRNAs are associated with the expression of PD-L1 or PD-1. In various tumor models, a majority of the identified miRNAs have demonstrated the ability to down-regulate the expression of PD-L1. For instance, in non-small cell lung cancer miR-140 (119), miR-142, and miR-197 have been observed to down-regulate PD-L1 expression, potentially attenuating its inhibitory effects on immune responses (120, 121). On the other hand, it is interesting to note that miR-3127-5p, in contrast, has been found to up-regulate PD-L1 expression (97). Some studies demonstrate the diverse roles of different miRNAs in regulating PD-L1 expression in colorectal cancer, with some miRNAs acting as suppressors and others as enhancers of PD-L1 expression through distinct molecular mechanisms. In colorectal cancer the miR-200 family, encompassing miR-200a, miR-200b, miR-429 and miR-200c act as inhibitors of PD-L1 expression. On the contrary, miR-20b, miR-21 and miR-130b have been reported to elevate PD-L1 expression in colorectal cancer by inhibiting the expression of PTEN (phosphatase and tensin homolog) (122, 123). Other miRNA (e.g., miR-138-5p) are also involved in the regulation of CTLA-4 (109). More miRNAs are yet to be discovered in relation to PD-1/PD-L1 and the exact mechanisms need to be further investigated. It is encouraging that our previous research found that NamiRNAs can regulate kidney cancer development by targeting enhancers (21). As to whether NamiRNAs play positive functions on their target genes by binding to the corresponding enhancers involved in immune therapy resistance for ccRCC remains to be eclucidated. Interestingly, our recent work suggests that miR-93-5p upregulates its target gene *MCM7* in ccRCC to evade an immune response (**Figure 6**).

## Discussion

Initially, tumorigenesis was believed to be primarily promoted via genetic alterations. However, it is now widely recognized that epigenetic factors play a critical role in the initiation and progression of cancers. Epigenetic features, including DNA methylations and histone modifications, should lead to altered gene expression patterns and contribute to cancer progression. Targeting these epigenetic aberrations appears to be a potential method in cancer therapy due to several reasons (124). First, cancers frequently exhibit dysregulation of epigenetic characteristics and recurrent mutations of epigenetic modulators. Second, unlike hereditary changes, epigenetic modifications can often be reversed. Third, it is possible to target enzymes or chromatin-binding proteins that control epigenetic traits. As a result, oncogenic epigenetic regulators can be suppressed, and normal epigenetic features can be restored to create epigenetic medications that can treat cancer. Although many epigenetic agents have been approved and have demonstrated some benefits over conventional chemotherapy drugs, there are still many problems that need to be resolved (125). For instance, epigenetic agents mostly target blood malignancies, and further research is still needed to better treat solid tumors and other challenging and complex disorders. Additionally, the majority of epigenetic changes are loss of function mutations, which are challenging to treat (126). Thus, target specificity of epigenetic agents remain to be resolved. In the future, one of the most important approaches for applying such medications may be to create pharmaceuticals that can interfere with the specific target.

Immune cells play a crucial role in anti-tumor therapy, and epigenetics regulate the differentiation, development, and function of various immune cells. More and more epigenetic drugs targeting immune cell surface receptors have been developed. Therefore, studying the regulation of epigenetics on immune cells is a key factor in the treatment of tumors. Moreover, we described how epigenetics regulates the differentiation and plasticity of CD4^+^, CD8^+^, T_regs_ and other immune cells. Although there are many related studies on the regulatory molecular mechanisms of epigenetics in regulating immune cell function, there are still many issues that are not very clear. For example, how do epigenetic molecular mechanisms regulate T cell differentiation into other subsets of T cell, and how do they control T cell differentiation and function? How do these epigenetic modifications play a role in anti-tumor immunity?

As an emerging class of immune modulators, epigenetic regulation plays a critical role in both facilitating the anti-cancer immune response and enabling tumor cells to evade immunosurveillance. Understanding and targeting these epigenetic mechanisms hold great promise for developing effective immunotherapies and overcoming immune evasion strategies employed by cancer cells. Epigenetic drugs not only have potential as standalone therapies, but may also be used in combination with ICIs to synergistically enhance the immune response by modulating gene expression and reversing immune suppression within the tumor microenvironment. This combined strategy has demonstrated potential in both preclinical research and clinical trials, offering new avenues for improving treatment outcomes. Here, we described how ICIs have achieved very encouraging results in cancer treatment in recent years. However, long-term clinical effectiveness of ICIs has been compromised by the development of primary and acquired drug resistance, and the adverse events associated with ICIs have not been extensively studied. Epigenetic drugs in contrast play an important role in the immune tumor microenvironment and may also reduce resistance to ICIs, thereby improving remission rates. Epigenetic therapy also faces challenges and limitations in the treatment of cancer, and it may exhibit varying specificity towards different tumor cell subtypes. For example, a particular epigenetic therapy might be effective in non-small cell lung cancer (NSCLC) but have limited effectiveness in small cell lung cancer (SCLC) (127). Future research needs to encompass a broader exploration of epigenetic changes within different subtypes to develop more specific treatment approaches. Overall, the combination of these two drugs represents a promising strategy that takes advantage of their synergistic effects to improve treatment outcomes in both solid tumors and hematological tumors. And, identifying the most effective biologic dosing strategies may hold the key to the success of these combined therapeutic approaches. Nevertheless, the specific cellular and molecular mechanisms that account for the superior effectiveness observed in combination therapy compared to monotherapy-driven anti-tumor effects are yet to be fully understood. Defining biomarkers that help identify patients suitable for treatment and monitor treatment responses is also a future challenge. Numerous therapeutic trials combining these two treatments are currently underway; however, the majority of them are in the initial phases of development (112, 128, 129), Moreover, other epigenetic mechanisms, such as long non-coding RNA (lncRNA) or miRNA, are not yet clinically targetable at present. The next significant advancements are expected to occur through investment in Phase 3 clinical trials. These trials are pivotal in evaluating the safety and effectiveness of prospective treatments, and their successful completion can pave the way for regulatory approval and widespread implementation.

## Author contributions

YL: Conceptualization, Funding acquisition, Supervision, Writing – original draft, Writing – review & editing. LW: Conceptualization, Supervision, Writing – original draft, Writing – review & editing. PM: Software, Resources. DJ: Data curation, Methodology, Supervision, Writing – review & editing. MZ: Supervision, Writing – review & editing. YS: Conceptualization, Funding acquisition, Supervision, Writing – original draft, Writing – review & editing.
